# Natural product FZJDXJ enhances CD8+ T-cell glycolysis to relieve exhaustion and augment antitumor immunity in HBV^+^ hepatocellular carcinoma

**DOI:** 10.3389/fnut.2026.1829200

**Published:** 2026-06-12

**Authors:** Fengna Yan, Yuqing Xie, Ke Shi, Lihua Yu, Qun Zhang, Zhiyun Yang, Xianbo Wang

**Affiliations:** 1 National Key Laboratory of Intelligent Tracking and Forecasting for Infectious Diseases, Beijing Ditan Hospital, Capital Medical University, Beijing, China; 2 Beijing Institute of Infectious Diseases, Beijing, China; 3 National Center for Infectious Diseases, Beijing Ditan Hospital, Capital Medical University, Beijing, China; 4 Beijing Key Laboratory of Viral Infectious Diseases, Beijing Ditan Hospital, Capital Medical University, Beijing, China

**Keywords:** CD8+ T cell immunosuppression, Fuzheng Jiedu Xiaoji Formula, glycolysis metabolomic, hepatocellular carcinoma, immune response modulation

## Abstract

**Objective:**

To investigate whether Fuzheng Jiedu Xiaoji Formula (FZJDXJ) reverses immunosuppression in HBV^+^ hepatocellular carcinoma (HCC) by reprogramming CD8^+^ T-cell glycolysis and enhance antitumor immunity.

**Methods:**

A translational study was conducted with 80 HBV^+^ HCC patients, Hepa1-6 tumor-bearing mouse models and *in vitro* CD8^+^ T-cell assays. Flow cytometry, GC/LC-MS metabolomics, Western blot and functional experiments were used to detect CD8^+^ T-cell immunophenotype, glycolytic metabolism and antitumor activity. A 1-year overall survival (OS) prediction framework was constructed and validated.

**Results:**

FZJDXJ increased peripheral CD8^+^ T-cell frequency, downregulated exhaustion markers (PD-1/CTLA-4/TIM-3) and upregulated glycolysis-related metabolites in patients, with metabolites correlating with CD8^+^ T-cell function. In mice, FZJDXJ suppressed tumor growth, enhanced tumor-infiltrating CD8^+^ T-cell cytotoxicity and glycolytic flux (GLUT1/HK2/PFKFB3/PKM2 upregulation). *In vitro*, FZJDXJ-containing serum dose-dependently promoted CD8^+^ T-cell glucose uptake and glycolytic molecule expression. The OS prediction framework had high discriminatory power (AUC=0.946). No severe safety signals were observed.

**Conclusion:**

FZJDXJ alleviates CD8^+^ T-cell exhaustion by enhancing glycolysis to strengthen antitumor immunity in HBV^+^ HCC. Immunometabolism is a promising target for herbal interventions, and glycolysis-immune features may serve as prognostic stratifiers. Prospective trials and ICI combination studies are warranted.

## 1 Introduction

Hepatocellular carcinoma is the third leading cause of cancer-related deaths globally, with a low 5-year survival rate (1). The high number of deaths (830,000 per year) emphasizes the poor prognosis of this disease (2). The development of HCC is influenced by various factors, such as viral infection, alcohol consumption, and nonalcoholic fatty liver disease. In recent years, several treatment options have been available for HCC patients, including liver transplantation, surgical resection, percutaneous ablation, radiotherapy, and new approaches like molecular targeting and immune checkpoint inhibitors (ICI). These advancements have brought hope for the survival of patients. However, despite these options, the 5-year survival rate remains at around 14%, and the treatment outcomes are not satisfactory (3, 4).

Dual infection with HBV and tumors leads to functional exhaustion of antigen-specific T-cells, characterized by increased expression of co-inhibitory molecules, weakened effector function, and loss of memory potential (5–7). Immune checkpoint molecules like PD-1 and CTLA-4 have been used to reinvigorate T-cell failure in tumors like HCC. However, the recovery of immune cells is short-lasting, and there are still limitations (6). In recent years, increased evidence has pointed out the potential of triggering the cross-talk between glycolytic metabolism and T-cell-mediated immune effects as a therapeutic target to reverse T-cell exhaustion (8, 9). Energy production and cell function are closely related to metabolism. Glucose metabolism is the primary process in tumor-infiltrated immune cells, which convert glucose into pyruvate through glycolytic enzymes. This pyruvate is further converted to lactic acid, producing ATP that is essential for immune cell activation, proliferation, and effector function (10, 11). Therefore, exploring new mechanisms of T-cell exhaustion, particularly those related to metabolism or metabolic pathways, can provide insights into potential therapeutic targets.

Traditional Chinese medicine (TCM) is a valuable cultural asset in China. Its unique features, such as multi-channel and multi-target treatment, have shown significant advantages in correcting tumor immune defects and improving overall immune function (12, 13). Fuzheng Jiedu Xiaoji Formula (FZJDXJ) is a classic prescription that has been used for several decades in the treatment of HCC. Our previous research has confirmed that FZJDXJ can inhibit disease progression in HCC patients by targeting the AKT/CyclinD1/p21/P27 pathway (14). While the principle of strengthening the spleen in prescription may contribute to its anti-tumor effects by enhancing anti-tumor immunity, its precise immune effects molecular mechanisms remain unclear. Therefore, we investigate whether FZJDXJ can reverse CD8+T cells dysfunction in HCC patients by competing the glycolytic metabolic pathway.

## 2 Materials and methods

The study was approved by the ethics committee of Beijing Ditan Hospital, Capital Medical University (license number: DTEC-KY2024-032-02). It was conducted in compliance with the Declaration of Helsinki.

### 2.1 The study population

This retrospective and ongoing study comprised 80 HBV-HCC patients. These individuals were admitted to the Center of Integrated Traditional Chinese and Western Medicine at Beijing Ditan Hospital affiliated to Capital Medical University, from November 2021 to January 2022. Inclusion criteria were: (i) Meeting Western diagnostic criteria for primary liver cancer; (ii) Having a history of chronic hepatitis B with HBsAg positivity for more than 6 months; (iii) Age ranging from 18 to 75 years; In addition, (iv) Maintaining oral treatment with FZJDXJ for over 3 month. Exclusion criteria were: (i) Diagnosis of cholangiocarcinoma; (ii) Concurrent complications with other tumor types; (iii) Incomplete clinical data. The enrolled patients were categorized into three groups: HBV-HCC group, palliative treatment (*n* = 30), FZJDXJ group, palliative therapy combined with FZJDXJ treatment (*n* = 30), ICI group, palliative therapy combined with ICI treatment (*n* = 20).

Regarding the diagnostic criteria for HCC, most patients received a histopathological diagnosis, while some were diagnosed clinically. Clinical diagnosis involved utilizing a minimum of two imaging methods (hepatic angiography, magnetic resonance imaging, computed tomography, liver ultrasound) to visualize distinct lesions. Alternatively, the diagnosis could be made with one imaging method displaying clear lesions along with an α-Fetoprotein (AFP) level ≥ 400 ng/ml^5^. Table 1 provides a summary of the clinical and demographic characteristics of all patients.

**Table 1:** Demographic and clinical characteristics of patients with HCC (*n* = 30), FZJDXJ (*n* = 30), ICI (*n* = 20).

	HBV-HCC *n* = 30 (%)	FZJDXJ *n* = 30 (%)	ICI *n* = 20 (%)	*P*-values
Patients background				
Age (mean ± SD)	58.07 ± 8.90	60.79 ± 10.77	57.18 ± 9.38	0.546
Gender (male/female)	24/6 (80/20)	26/4 (86.67/13.33)	11/9 (55/45)	0.665
Hypersplenism	16/14 (53.33/46.67)	21/9 (70/30)	11/9 (55/45)	0.987
PVTT	17/13 (56.67/43.33)	9/11 (63.33/36.67)	15/5 (75/25)	0.270
HBV-related indicators				
HBV DNA (positive/negative)	8/22 (26.67/73.33)	5/25 (16.67/83.33)	8/12 (40/60)	0.490
HBeAg ≥ 1 (IU/ml), *n* (%)	11/18 (36.67, 60)	11/18 (36.67, 60)	17/3 (85, 15)	0.117
Laboratory data				
WBC ≤ 4 × 10^9^/L, *n* (%)	19/11 (63.33, 36.67)	8/22 (26.67, 73.33)	14/6 (70, 30)	0.149
NLR	3.21 (2.38, 5.58)	2.35 (1.83, 2.96)	4.8 (4.24, 10.53)	0.005
PLT ≤ 100 × 10^9^/L, *n* (%)	19/11 (63.33, 36.67)	17/12 (56.67, 40)	12/8 (20, 80)	0.099
AST ≥ 40 (U/L), *n* (%)	16/13 (53.33, 43.33)	12/17 (40, 56.67)	5/15 (25, 75)	0.128
ALT ≥ 50 (U/L), *n* (%)	21/8 (770, 26.67)	11/18 (36.67, 60)	8/12 (40, 60)	0.614
ALB ≤ 40 (g/L), *n* (%)	6/23 (20, 76.67)	17/12 (56.67, 40)	12/8 (20, 80)	0.377
TBIL ≥ 18.8 (μmol/L), *n* (%)	10/19 (52.63, 47.37)	10/19 (33.33, 63.33)	6/14 (30, 70)	0.306
GGT ≥ 60 (U/L), *n* (%)	13/16 (43.33, 53.33)	12/7 (63.16, 36.84)	5/15 (25, 75)	0.459
CRP ≥ 5 (mg/L), *n* (%)	14/12 (46.67, 40)	12/13 (40, 43.33)	6/14 (30, 70)	0.105
LDH ≥ 250 (U/L), *n* (%)	18/10 (60, 33.33)	11/18 (36.67, 60)	6/13 (30, 65)	0.871
Tumor-related indicators				
AFP ≥ 8.8(ng/ml)	11/18(37.93, 62.07)	10/9(52.63, 47.37)	10/10(50/50)	0.362
Tumor solitary (solitary/multiple)	11/18(37.9/62.1)	14/15(26.3/73.7)	5/15(25%75%)	0.428
Tumor size, cm, (< 5/>5)	16/13(53.33/43.33)	15/14(50/46.7)	6/14(30/70)	0.206
Child grade A/B/C	7/11/12(23.33/36.67/40)	10/14/6(33.33/46.67/20)	5/10/15(25/50/75)	0.107
Types of immune checkpoint inhibitors (Sintilimab/ Tislelizumab/Camrelizumab)			10/9/1	

Values are represented as mean (±SD) or %. P values. *P* < 0.05 comparison between HCC considered statistically significant . SD, standard deviation; NLR, Neutrophil-lymphocyte ratio; ALT, alanine aminotransferase; AST, aspartate aminotransferase; γ-GGT, γ-glutamyl transferase; AFP, alpha-fetoprotein; CRP, C reactive protein; ALB albumin; HBeAg, hepatitis B e antigen; TBIL, total bilirubin; HBsAg, hepatitis B s antigen; CREA, creatinine; LDH, lactate dehydrogenase; TG, triglyceride.

FZJDXJ treatment was defined based on medical records indicating TCM usage for a minimum of 7 days during the hospitalization and at least 3 month before admission. The herbal formula FZJDXJ consists of 10 herbs: *Paris polyphylla* (15 g), *Ophiopogon japonicus* (15 g), *Hedysarum multijugum maxim* (15 g), *Curcumae rhizoma* (15 g), *Codonopsis radix* (15 g), *Angelicae sinensis radix* (15 g), *Angelica sinensis* (15 g), *Atractylodis macrocephatae* (15 g), *Rehmanniae radix praeparata* (15 g), *Poria cocos* (*Schw.) wolf* (15 g), *Pinellia ternata* (9 g). Previous studies have identified 1,619 active ingredients of FZJDXJ using HPLC-MS/MS. All drugs are uniformly boiled and provided by the Chinese pharmacy of Beijing Ditan Hospital (14).

### 2.2 Sample collection

Upon admission, 10 ml of peripheral blood samples were collected. Peripheral blood mononuclear cells (PBMC) and serum were separated as previously described (15).

### 2.3 Flow cytometry staining and analysis

Antibodies used were as follows: BD: anti-CD3 (SK7, catalog number 563800), anti-PD-1 (EH12.1, catalog number 564017), anti-TIM-3 (7D3, catalog number 565564), anti-GLUT1 (202915, catalog number 566580). Biolegend: anti-CD4 (SK3, catalog number 344638), anti-CD8 (SK1, catalog number 344732), PE-CF594-conjugated anti-CD25, PE-conjugated anti-CD127, anti-CD45RA (HI100, catalog number 304120), anti-CCR7 (G043H7, catalog number 353208), anti-CD38 (HB-7, catalog number 356610), anti-HLA-DR (L243, catalog number 307640), anti-CD152 (L3D10, catalog number 349908), eBioscience: anti-TIGIT (MBSA43, catalog number 25-9500-42**)**. abcam: anti-HK2 (catalog number 237314). Novus: (catalog number NBP2-53421F) and the corresponding isotype controls.

Stain extracellular antibodies from peripheral blood single cell suspension (approximately 1 million cells per milliliter), incubate at room temperature and dark for 30 min. Then, the cells in a solution containing 40 μM 2NBDG (catalog number NBP2-53421F; eBioscience) or 0.4 μM serum-free RPMI of M glucose maintain at 37 °C for 30 min. Data were collected utilizing an LSR Fortessa flow cytometer and processed utilizing FlowJo software (Tree Star, Inc., Ashland, OR, USA).

### 2.4 Metabolomic profiling

All chemicals and solvents were of analytical or HPLC grade. Water, methanol, acetonitrile, and formic acid were procured from Thermo Fisher Scientific (Waltham, MA, USA). Pyridine, n-hexane, methoxylamine hydrochloride (97%), and N, O-bis (trimethylsilyl) trifluoroacetamide (BSTFA) with 1% Trimethylchlorosilane (TMCS) were sourced from CNW Technologies GmbH (Düsseldorf, Germany). L-2-chlorophenylalanine was obtained from Shanghai Hengchuang Bio-technology Co., Ltd (Shanghai, China).

The frozen mouse tumor tissue and patient serum samples were thawed to room temperature. Specifically, 100 μl of each sample was combined with 300 μl of L-2-chlorophenylalanine (0.3 mg/ml) dissolved in methanol, serving as an internal standard. The resulting mixture was vortexed for 10 s. Subsequently, 300 μl of an ice-cold mixture of methanol and acetonitrile (2:1, vol/vol) was added, and the mixtures were vortexed for 1 min. The entire sample set underwent ultrasonic extraction for 10 min within an ice-water bath, followed by storage at−20 °C for 30 min. After extraction, the mixture was centrifuged at 4 °C (13,000 rpm) for 10 min. A supernatant volume of 0.15 ml was transferred to a glass vial and subjected to freeze concentration using a centrifugal dryer. A quality control (QC) sample was prepared by combining aliquots of all the samples to create a pooled sample. A 150 μl aliquot of the supernatant was transferred to a glass sampling vial and vacuum-dried at room temperature. Subsequently, 80 μl of methoxylamine hydrochloride (15 mg/ml) in pyridine was added, and the resulting mixture was vigorously vortexed for 2 min. The mixture was then incubated at 37 °C for 90 min. Following this, 50 μl of BSTFA (with 1% TMCS) and 20 μl of n-hexane were added to the mixture, which was again vigorously vortexed for 2 min and then derivatized at 70 °C for 60 min. The samples were left at room temperature for 30 min prior to GC-MS analysis. To each sample, 300 μl of a methanol and water mixture (1/4, vol/vol) was added. The samples were then vortexed for 30 s and extracted by ultrasonication for 3 min in an ice-water bath. Subsequently, the samples were placed at −20 °C for 2 h. Afterward, the samples were centrifuged at 4 °C (13,000 rpm) for 10 min. The resulting supernatants were collected using crystal syringes with a volume of 150 μl from each tube. These supernatants were then filtered through 0.22 μm microfilters and transferred to LC vials. The vials were kept at −80 °C until LC-MS analysis.

### 2.5 FZJDXJ tumor transplantation model

The animal use protocol has been reviewed and approved by the Animal Care and Use Committee of Capital Medical University (license number: BJTCM-M-2022-04-01). 30 specific pathogen-free (SPF) male mice C57/BL6 mice (aged 6–8 weeks, weighing 18 ± 2 g; Beijing Sbeifu Biotechnology Co., Ltd., Beijing, China) were given *ad libitum* access to food and water for 1-week adaptive feeding period. Subsequently, subcutaneous transplantation of the mouse HCC cell line Hepa1-6 cells (1 × 10^7^ cells/mouse; CCTCC number: 3142C0001000000339) was performed into the right axillary region of each mouse. One week was allowed for the tumors to establish. Once the tumors reached a size of 50 mm, they were orally administered 215 mg/kg of FZJDXJ treatment once daily for 14 days, continuously (3). We have previously published that a dose of 215 mg/kg has the greatest anti-tumor effect and no significant toxicity, therefore it is defined as the optimal dose (14). The specific calculation method is selected based on the conversion of the body surface area of the human clinical dose (equivalent to the clinical oral administration of 70 kg adult), We performed a pre-experiment with gradient doses (107.5, 215, 430 mg/kg) to evaluate tumor inhibition rate, mouse body weight, and liver/kidney function. Mice were intraperitoneally injected with 10 mg/kg of an anti-mouse PD-1 monoclonal antibody as a positive control group (InvivoMAb anti-mouse PD-1 (CD279), Cat# BP0146, Clone: RMP1-14, BioXcell, USA). For the CD8+ T cell depletion experiment, mice were treated with 10 mg/kg of anti-mouse CD8a antibodies (InvivoMAb anti-mouse CD8, Cat # BP0117, Clone: 2.43, BioXcell, USA). The control group of mice received the same volume of Phosphate-buffered saline (PBS). Great efforts were made to minimize the number of animals used in the experiments and their discomfort. Throughout the animal experiment, mice were injected five times at 3-day intervals with anti-mouse PD-1 monoclonal antibody or CD8a antibodies, and tumor diameters were routinely measured utilizing a caliper. Tumor volume was estimated using the formula: length × width^2^ × 0.5. FZJDXJ treatment was administered continuously during the animal experiment.

### 2.6 Western blot analysis

Total protein was extracted from 30 mg of tumor tissue and subsequently subjected to denaturing sodium dodecyl sulfate-polyacrylamide gel electrophoresis (SDS-PAGE). The separated proteins were then transferred onto a polyvinylidene fluoride membrane (MilliporeSigma, Burlington, MA, USA). The membrane was blocked using 5% milk for followed by overnight incubation at 4 °C with primary antibodies: anti-pyruvate kinase (PKM2; 1:1000, CST, mAb #4053), fructose bisphosphatase-2 isozyme 3 (PFKFB3; 1:1000, CST, mAb #13123), hexokinase-2 (HK2; 1:300, Abcam, #ab209847), glucose transporters (GLUT1; 1:300, Abcam, #ab115730), and β-actin (Proteintech Group, Rosemont, IL, USA; Biotin-60008). After three washes with 1 × TBST, the membrane was incubated with secondary antibodies for 1.5 h. The secondary antibodies utilized were goat anti-rabbit IgG-HRP (Immunoway, RS0002) and goat anti-mouse IgG-HRP (RS0001), both diluted at 1:10,000. The intensity of bands was quantified utilizing Image J software (version 1.52) and normalized to beta-actin levels. The original images can be referred to in the accompanying source data file.

### 2.7 Isolation of mononuclear cells from tumor tissues and flow cytometry

The procedure for isolating tumor-infiltrating T cells was consistent with the previously described method involving staining with fluorescent-conjugated monoclonal antibodies (16). The antibodies utilized were as follows: anti-mouse APC-H7-conjugated anti-CD45, anti-mouse BV786-conjugated anti-CD3, FITC-conjugated anti-CD4, BV510-conjugated anti-CD8, BV711-conjugated anti-PD-1, BV650-conjugated anti-Tim-3, APC-conjugated anti-CTLA-4, Pe-cy7-conjugated anti-TIGIT, PE-CF594-conjugated anti-CD69, BV605-conjugated anti-CD103, AF700-conjugated anti-Granzyme B, FITC-conjugated anti-Ki67, and APC-conjugated anti-Perforin. Corresponding homotypic controls were employed for comparison.

### 2.8 Bioinformatic and statistical analysis

The original GC/LC-MS data were subjected to processing using Progenesis QI V2.3 software (Nonlinear Dynamics, Newcastle, UK). This processing encompassed baseline filtering, peak identification, integration, retention time correction, peak alignment, and normalization. Key parameters included a 5 ppm precursor tolerance, 10 ppm product tolerance, and a 5% production threshold. Compound identification relied on the precise mass-to-charge ratio (M/z), secondary fragments, and isotopic distribution. The Human Metabolome Database (HMDB), Lipidmaps database (version 2.3, Lipid Maps Initiative, UC San Diego, La Jolla, CA, USA), Metlin, Electron Microscopy Data Bank (EMDB), Protein Model Database (PMDB), and self-constructed databases were employed for qualitative analysis.

The data matrix was imported into R for Principle Component Analysis (PCA) to assess the overall distribution among the samples and the stability of the analysis process (17). Additionally, Orthogonal Partial Least-Squares-Discriminant Analysis (OPLS-DA) and Partial Least-Squares-Discriminant Analysis (PLS-DA) were employed to identify the group metabolites that exhibit differences. We used random forest combined with recursive feature elimination (RFE) for feature selection and finally identified three core variables integrating clinical, immune, and metabolic parameters. Seven-fold cross-validation and 200 permutation tests were applied to avoid overfitting.

GraphPad Prism version 5.0 (GraphPad Software, Inc., San Diego, CA, USA) and SPSS version 19.0 (IBM Corp., Armonk, NY, USA) were utilized to analyze baseline statistics and polychromatic flow staining results in clinical patients. Mean ± standard deviation (SD) was utilized to present consistent quantitative data, and these data were subjected to *t*-test analysis (18). Non-normally distributed data were represented as the median of the quartile range and analyzed utilizing the Mann–Whitney *U*-test (18). Pearson's correlation coefficient was utilized to assess the correlation between normally distributed data, while Spearman's correlation coefficient was employed for non-normally distributed data. Statistical significance was determined at *P* < 0.05 (18).

## 3 Results

### 3.1 Baseline characteristics

Throughout the study duration, 80 patients diagnosed with HBV-HCC were enrolled. Among these, 30 patients received FZJDXJ treatment for over 3 month, 30 had advanced HBV-HCC, and 20 underwent ICI treatment (at least three courses). All included indicators are well-recognized clinical and prognostic factors for HBV+HCC, and their selection is closely associated with the study theme of FZJDXJ regulating immune metabolism to enhance anti-tumor immunity in HBV+HCC patients. Comparison between three groups revealed no significant disparities in terms of age, gender, liver function (Child grade), HBV-DNA replication, neutrophil-to-lymphocyte ratio (NLR), platelet count, aspartate aminotransferase (AST), total bilirubin (TBIL), gamma-glutamyltransferase (GGT), C-reactive protein (CRP), and tumor characteristics (Table 1). We confirmed that NLR differed significantly among the three groups (*P* = 0.005). Multivariate logistic and Cox regression analyses were used to adjust for NLR, age, liver function, and other baseline variables. The main conclusions regarding CD8+T cell function, glycolytic metabolites, and prognosis remained unchanged after adjustment.

### 3.2 Baseline immune cell quantification and phenotype in peripheral blood

**Figure 1:**
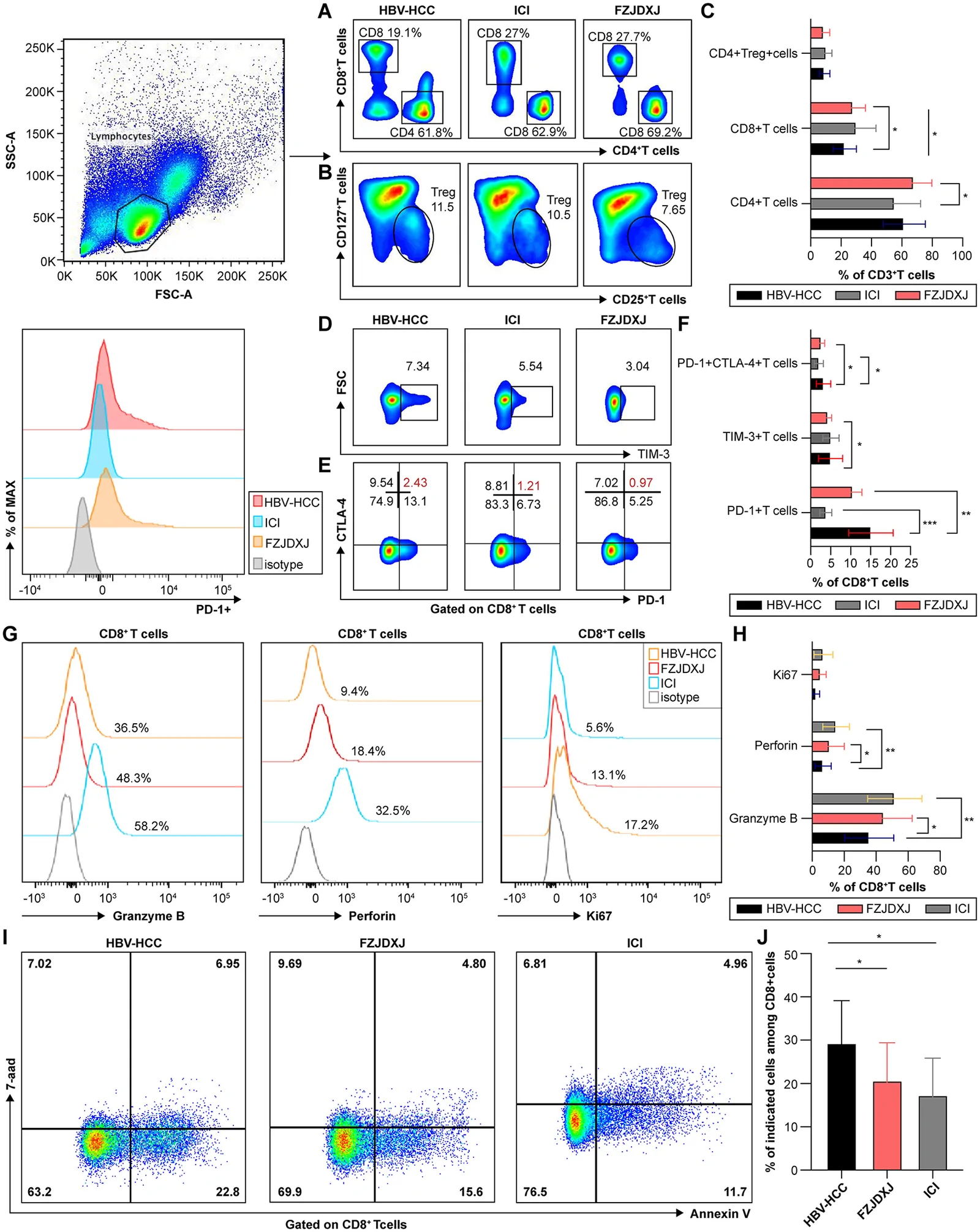
Quantification of anti-tumor immune effect phenotype in peripheral blood mononuclear cells (PBMCs) from HCC (*n* = 30), FZJDXJ (*n* = 30), ICI treatment (*n* = 20). **(A–C)** The percentage of CD4+T cells (CD3+CD4+), regulatory T cells (CD3+ CD4+ CD25^hi^ CD127^low^) and CD8+ T cells (CD3+CD8+).The expression of PD-1, Panels **(D–F)** show CTLA-4 and TIM-3, and panel **(G)** presents representative flow cytometry histograms depicting the cytotoxic function (granzyme B, perforin) and proliferative capacity (Ki67) of CD8^+^ T cells in the three groups; panel **(H)** shows the quantitative analysis of effector cytokines (IFN-γ and TNF-α) in CD8^+^ T cells, expressed as the percentage of cytokine-producing cells. Cell death (Annexin V;) **(I, J)** on CD8+T cells. *P-values* are calculated by one-way ANOVA for three-group comparison and Tukey's test for two-group comparison. *P < 0.05, **P < 0.01, ***P < 0.001.

HCC patients often experience a significant deficiency in tumor immunity. It is primarily characterized by CD8+T lymphocyte exhaustion, abnormal activation of Treg cells with immunosuppressive function, compromised tumor cell surveillance and the promotion of HCC progression (19, 20). To better understand how FZJDXJ treatment affects the peripheral immunosuppressive microenvironment in HBV-HCC patients. Flow cytometry analyzed that the proportions of CD4+ and CD8+ T cells obviously increased in peripheral blood with FZJDXJ treatment. While Treg cells did not show statistical significance, a downward trend was noticeable (Figures 1A–C). We analyzed no significant alterations with CD4+ T cell exhaustion phenotype, activation and differentiation of CD8+T cells following FZJDXJ treatment (*P* > 0.05; Supplementary Figures S1A–C). Furthermore, the expression of PD-1^+^, Tim-3^+^, and PD-1^+^CTLA-4^+^ on CD8^+^T cells remarkable decreased after FZJDXJ and ICI treatment (*P* < 0.05; Figures 1D–F). Additionally, FZJDXJ drove CD8+ T cell cytotoxic function and delayed cell death (Figures 1G–J). The FZJDXJ group significantly increased IFN-γ and TNF-α of CD8+T cells compared with the HBV-HCC group. The ICI group was used as a positive control and showed similar or stronger effects (Figure 1H).

### 3.3 Peripheral metabolic activity alterations in HBV-HCC patients with FZJDXJ treatment

**Figure 2:**
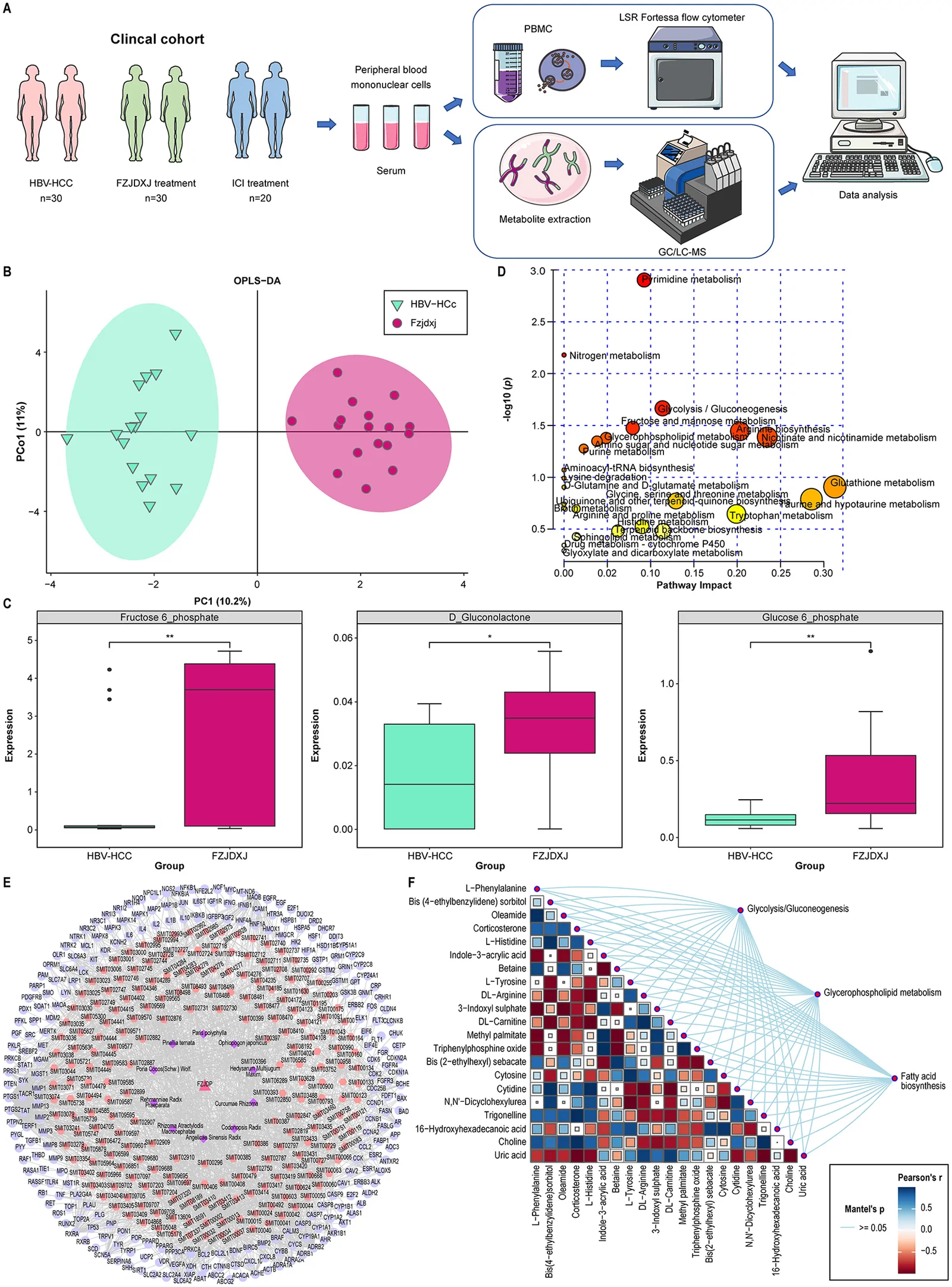
Metabolic characteristics of HBV-HCC patients with FZJDXJ treatment. **(A)** Study design and workflow of biomarker discovery in HCC (*n* = 30), FZJDXJ treatment (*n* = 30), ICI treatment (*n* = 20); **(B)** PCA, principal components analysis; OPLS-DA, orthogonal partial least squares showing comparisons of the HCC, ICI, FZJDXJ patients' metabolome profiles. **(C)** The histogram and violin map significantly differential metabolites for *P-value* and fold change value in HCC, FZJDXJ groups. Metabolites with VIP ≥ 1 and fold change ≥2 or fold change ≤ 0.5 were considered differential metabolites. **(D)** KEGG pathway enrichment analysis between the HCC and FZJDXJ treatment group by GC/LC-MS metabolomic analyses. Core sub-network of the FZJDXJ for the treatment of HCC. **(E)** Edges in the network represent interactions between targets, predicted targets using a blue circle. **(F)** Pearson correlation analysis of pharmacological indicators (Glycolysis/Gluconeogenesis, Glycerophospholipid metabolism, Fatty acid biosynthesis) and potential metabolites. The box chart shows the median ± quartile. *P < 0.05, **P < 0.01, ***P < 0.001.

To explore the metabolites of HBV-HCC patients treated with FZJDXJ, GC/LC-MS metabolomics analysis was conducted utilizing paired patient plasma samples. A concise workflow of the entire study design is shown in the Figure 2A. Totally, 80 plasma samples from patients in our study. Orthogonal Partial Least Squares Discriminant Analysis (OPLS-DA) was employed to visualize the overall distribution and discern distinct metabolite patterns among the groups. The outcomes indicated that samples from both HBV-HCC and FZJDXJ treatment groups exhibited tightly clustered positions, implying consistent and marked disparities in metabolite distribution among the samples (Figure 2B). GC/LC-MS platform analysis results found that the metabolites of Fructose 6 phosphate, D_Gluconolactone, Glucose 6_phosphate classified by glycolysis/gluconeogenesis were significantly increased (Figure 2C) with FZJDXJ treatment compared with HBV-HCC patients. At the same time, amino acid metabolites, Tetranor-(+)-S-145, Hydroxy acids and derivatives, Glyoxylic acid, alpha-Hydroxyisobutyric acid, S-(PGJ2)-glutathione, Sodium Tetradecyl Sulfate, S-(PGJ2)-glutathione, N-carbomoylglycine classified by hydroxy acids were elevated (Supplementary Figures S2A–C). Kyoto Encyclopedia of Genes and Genomes (KEGG) pathway enrichment analysis of differential metabolites showed that Glycolysis/gluconeogenesis, Fructose and mannose metabolism, Pantothenate and CoA biosynthesis had the highest risk factor (Figure 2D). To construct the component target network, 1,619 active compounds identified by our previous HPLC-MS/MS analysis were screened using the Traditional Chinese Medicine Systems Pharmacology Database and Analysis Platform (TCMSP) and Bioinformatics Analysis Tool for Molecular mechANism of Traditional Chinese Medicine (BATMAN-TCM) databases with thresholds of oral bioavailability (OB) ≥ 30% and drug-likeness (DL) ≥ 0.18. Corresponding targets with a confidence score ≥ 0.7 were retained, and HCC-related targets were obtained from DisGeNET and STRING, yielding 148 intersecting targets. The component-metabolite-gene-network was constructed as shown in the Figure 2E. The network comprised 98 differential metabolites and 148 HCC components. Among these network, the key enzyme in glycolysis, phosphofructokinase, liver type (PFKL) and pyruvate kinase L/R (PKLR) jointly identified using an integrated metabolomics and network pharmacology approach may play an essential role in the therapeutic effect of FZJDXJ in HCC. The correlations between FZJDXJ phytochemicals and endogenous metabolites are summarized in the Figure 2F. Positive correlations indicate synergistic regulation of glycolysis; negative correlations reflect reduced immunosuppression. All components were positively (blue) or negatively (red) correlated with key FZJDXJ- ameliorated Glycolysis/Gluconeogenesis-related metabolites.

### 3.4 FZJDXJ treatment alters the glycolytic metabolic pathway of CD8+T cells and affects HCC patients' prognosis

**Figure 3:**
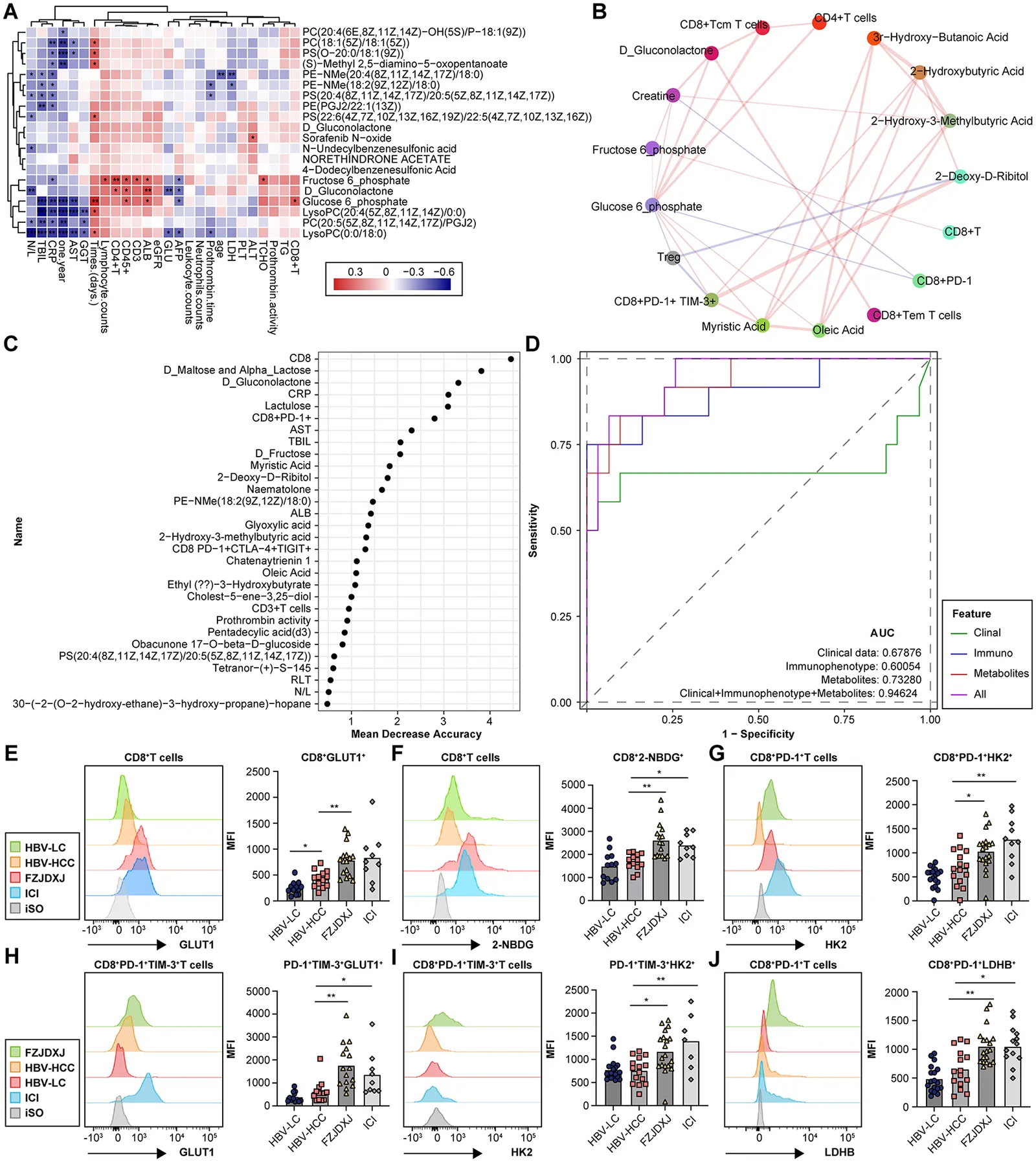
FZJDXJ treatment alters the glycolytic metabolic pathway of CD8+T cells and affects patient prognosis. **(A, B)** Heatmap of the relative abundance of 26 significantly different metabolites with CD4^+^, CD8^+^T cell immunosuppressive phenotypes and clinical indicators between HCC and FZJDXJ group. (*FDR < 0.05). The red squares indicate positive correlations, whereas the blue squares indicate negative correlations. **(C)** The random forest method was used to identify the markers of clinical data, immune markers, and metabolomics, which were closely related to survival prognosis. **(D)** The receiver operating characteristic (ROC) curve and its corresponding area under the curve (AUC) were established using the above sorted markers. (**E–J**) Flow cytometry analyzed the average fluorescence intensity (MFI) of key molecules in the glycolytic metabolic pathway. (**E, F**) Transporters GLUT1, 2-NBDG uptake on CD8+ T cells. (**G, J**) HK2 and LDHB on CD8+PD-1+T cell. **(H, I**) GLUT1, HK2 on CD8+PD-1+Tim-3+T cell in peripheral blood of HCC, FZJDXJ, and ICI treatment patients. *P < 0.05, **P < 0.01, ***P < 0.001 compared to HCC control.

The relationship between peripheral metabolites and clinical parameters was examined utilizing Spearman's correlation analysis. We observed that glycolysis metabolites, such as Fructose 6 phosphate, D_Gluconolactone, Glucose 6 phosphate, which were significantly increased with FZJDXJ treatment, showed a positive correlation with percentage of CD3^+^T, CD4^+^T, especially CD8^+^T cells (Figure 3A). To further characterize the metabolic-immune crosstalk in the tumor microenvironment, we constructed a correlation network integrating key metabolites and immune cell populations (Figure 3B). Notably, the immunosuppressive Treg cells showed significant negative correlations with the glycolytic intermediates glucose 6-phosphate and fructose 6-phosphate (*P* < 0.05). Similarly, exhausted CD8+PD-1+T cells were negatively correlated with 2-deoxy-D-ribitol, as well as with glucose 6-phosphate and fructose 6-phosphate. These negative correlations suggest that elevated levels of these metabolites may be associated with reduced abundance or function of immunosuppressive T cell subsets in our cohort. In contrast, most of the detected metabolites displayed predominantly positive correlations with effector and memory T cell populations (CD4+T cells, CD8+Tcm and Tem cells), highlighting a divergent pattern of metabolic regulation between immunosuppressive and effector T cell compartments. They exhibited a negative correlation with immunosuppressive phenotypes, Treg, and CD8+PD-1+ T cells. The metabolism of glycerophospholipids, lipids, steroids, steroid derivatives, and hydroxy acids exhibited no significant correlation with immune cells (Supplementary Figures S3A–C). Furthermore, a random forest analysis was performed, and an optimal classifier model was constructed using cross-validation. Thirty key indicators comprising clinical data, immune markers, and significant changes in metabolites were selected for distinguishing the best prognosis of HCC patients. The cohort was randomly split into a training set (*n* = 56, 70%) and a test set (*n* = 24, 30%). 100 bootstrap resampling were performed, and the Area under the curve (AUC) remained stable between 0.923 and 0.951, confirming model robustness. Among these key indicators, combining three was the most effective in differentiating 1-year mortality in HBV-HCC patients. The corresponding areas under the curve (AUC) for the four findings were 0.679, 0.600, 0.733 and 0.946, respectively (Figures 3C, D). Based on the metabolomics-based predictions, we validated and confirmed that the tumor microenvironment of HBV-HCC is associated with impaired glucose utilization and suppressed glycolytic metabolism in CD8+ T cells. FZJDXJ treatment significantly increase the level of 2-NBDG representing glucose uptake capacity on CD8+T cells. Increase the expression of GLUT1 and HK2 on CD8+T cell exhaustion population (PD-1+, PD-1+TIM-3) and restore the glucose transport ability, quickly initiate glycolytic metabolism to increase energy (Figures 3E–J).

### 3.5 FZJDXJ inhibits tumor growth, alleviates the exhaustion phenotype, and restores cytotoxicity of CD8+T cells in Hepa1-6 tumor-bearing mice

**Figure 4:**
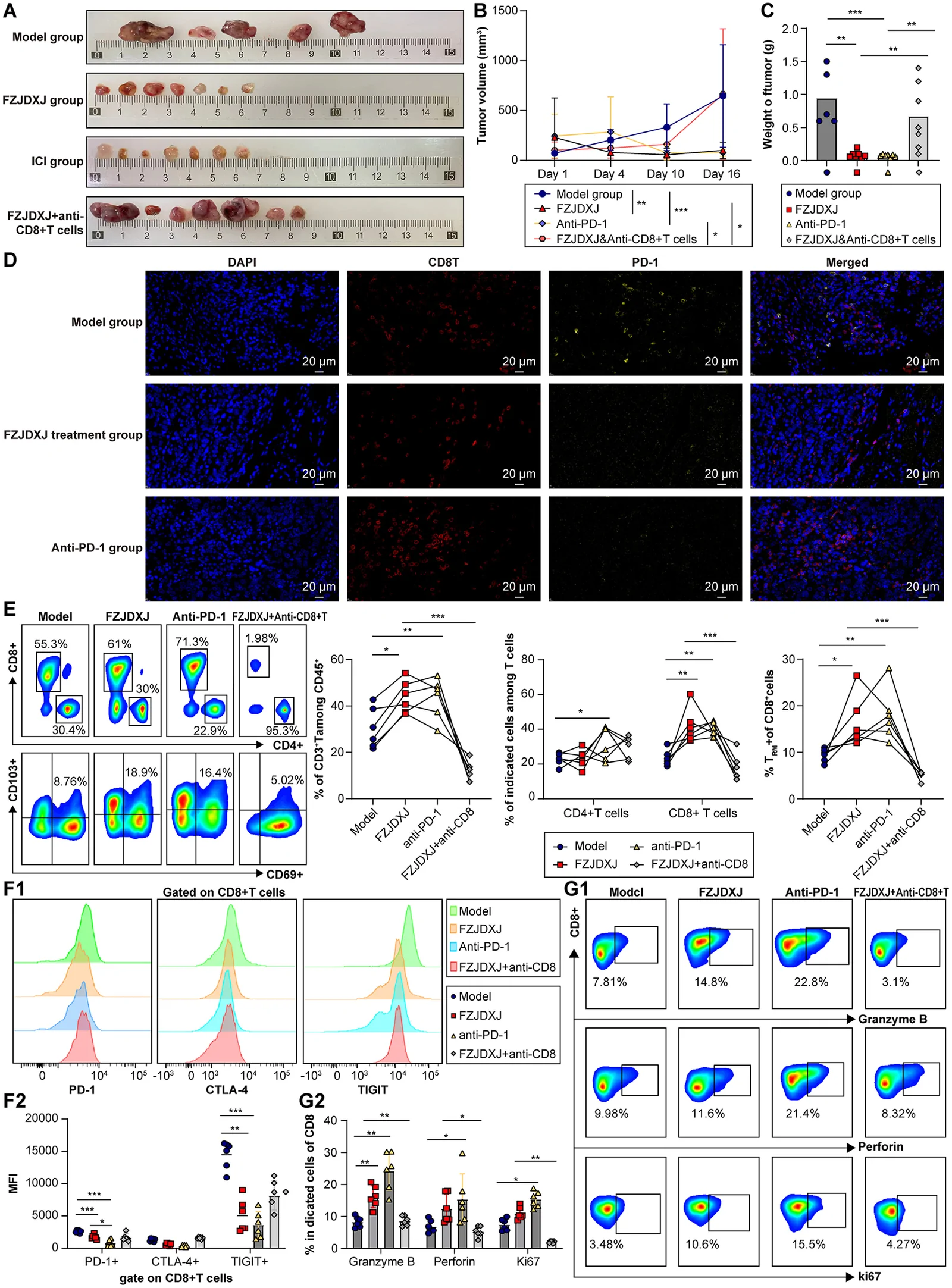
FZJDXJ therapy inhibits tumor growth of HCC and CD8+T cell immunosuppression *in vivo*. **(A**) Photographs of representative tumor blocks collected from mice from different treatment groups. (**B**) Tumor volume of Hepa1-6 tumor bearing-mice treated with ormal saline, FZJDXJ, anti-PD-1, FZJDXJ+anti-CD8+T (*n* = 7). (**C**) Tumor weight of each group for the last treatment. (**D**) Multiplexed IHC of Representative pictures were compared according to the sites of lesions of Hepa1-6 tumor mice. **(E)**: Representative flow cytometry plots **(left)** and corresponding quantitative analysis **(right)** showing the frequencies of tumor-infiltrating CD3^+^, CD8^+^, and TRM cells in the four groups. Data are presented as individual values with mean ± standard error of the mean (SEM). *P < 0.05, **P < 0.01, *P < 0.001. **(F1, F2)**: The percentage of PD-1^+^CD8^+^, TIGIT^+^CD8^+^, CTLA-4^+^CD8^+^T cells from tumor tissues of mice (*n* = 6). **(G1, G2)**: Tumor-infiltrating T cells (TIL) in each group described as in cytotoxicity function (Granzyme B, Perforin) and proliferation (Ki-67) and statistical analysis (*n* = 6). *P < 0.05, **P < 0.01, ***P < 0.001 compared to Hepa1-6 tumor mice control.

We conducted a comparative analysis of tumor and immune responses among various groups of mice. Our study revealed that FZJDXJ treatment substantially curtailed tumor growth (Figure 4). Both FZJDXJ and ICI treatments caused a notable reduction in tumor weight and volume compared to the model group (*P* < 0.05). However, the anti-tumor efficacy of FZJDXJ on solid tumors was found to be inferior to anti-PD-1 treatment (Figures 4A–C). FZJDXJ and anti-PD-1 treatment exhibited notable increase positive expression of tumor-infiltrating CD8^+^T cells compared with model mice. While the proportion of CD8^+^PD-1^+^ double-positive cells decreased (Figure 4D). Figure 4E (left subpanel) displays representative flow cytometry plots, while the right subpanel of Figure 4E presents the corresponding quantitative analysis results. The expression of co-inhibitory molecules, including PD-1, TIGIT, and CTLA-4, which are indicative of CD8^+^T cell exhaustion, notably decreased (Figure 4F). More significantly, we also observed that FZJDXJ treatment significantly restored CD8+T cell killing (Granzyme B, perforin) and proliferating ability (Ki67; Figure 4G). However, it is important to note that these effects were more obvious in the anti-PD-1 group. These findings indicated the definite efficacy of FZJDXJ in enhancing the anti-tumor immune response.

### 3.6 FZJDXJ enhances CD8+T cells glycolysis metabolic pathways in Hepa1-6 tumor-bearing mice

**Figure 5:**
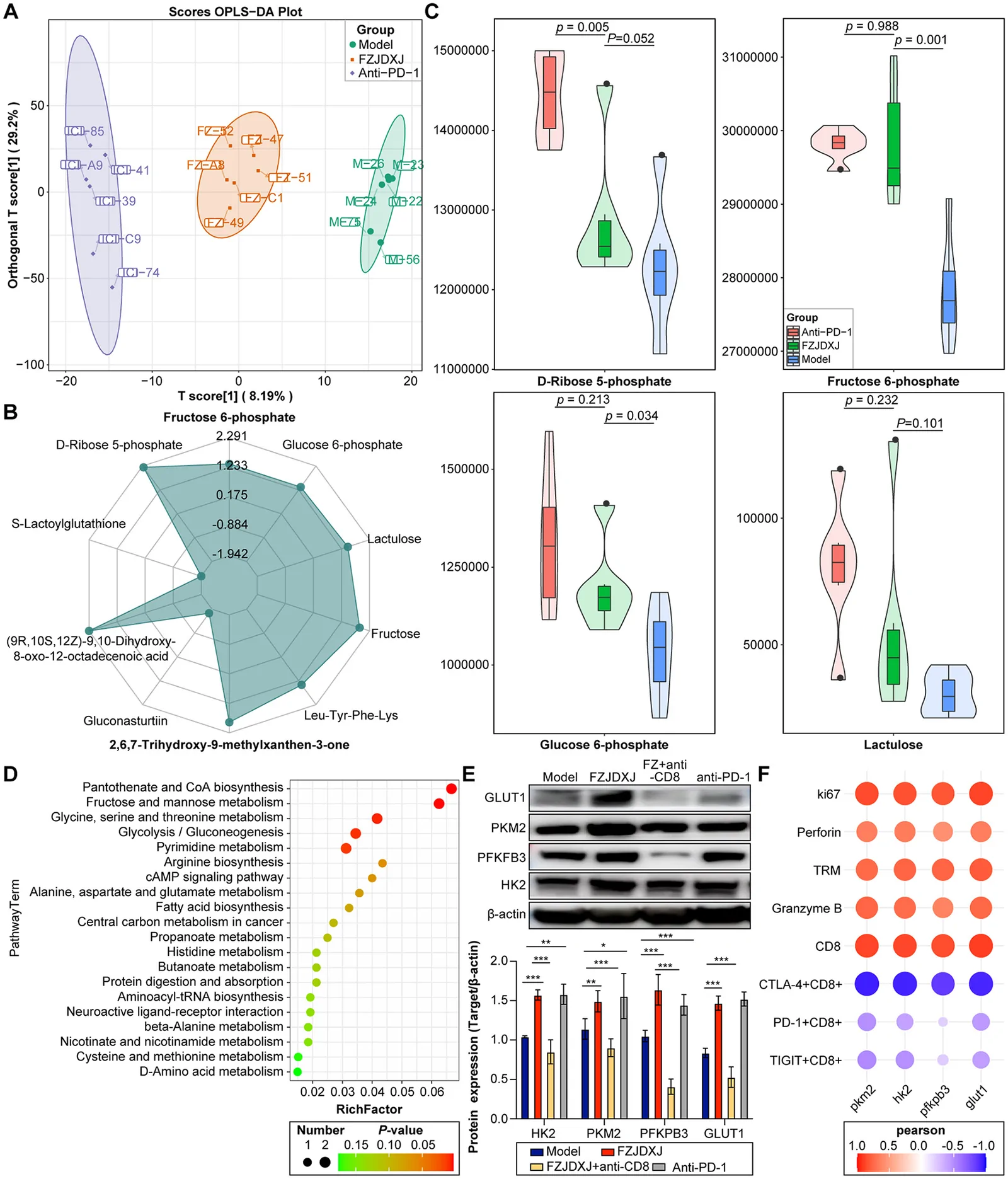
FZJDXJ promoted the CD8+T cells glycolytic metabolism and enhances the tumor killing function in Hepa1-6 tumor bearing mice. **(A)**: OPLS-DA analysis score showing metabolome profiles of the HCC, FZJDXJ, anti-PD-1 group mice. **(B)**: Radar map shows the quantitative results of metabolites with significant differences in the three groups, and the top 10 metabolites with the largest absolute value of log_2_FC are drawn for radar map. **(C)**: Violin map shows the quantitative analysis results of different products in the metabolic pathways of organic acids and glucose. **(D):** KEGG pathway enrichment analysis between the HCC and FZJDXJ treatment group by LC-MS/MS metabolomic analyses. Box and whisker plots show median ± quartiles (box), min/max (whiskers). **(E)** Western blotting results depicting the HK2, PKM2, PFKPB3, and GLUT1 protein expression in the tumor tissue. **(F)** The proportion of CD8^+^T cells and killing function was associated with different expression of glycolytic rate-limiting enzyme after FZJDXJ treatment. Spearman's grade correlation is used, with red indicating a strong positive correlation and green indicating a strong negative correlation, and the darker the color, the larger the absolute value of the correlation coefficient between samples. *P < 0.05, **P < 0.01, ***P < 0.001 compared to Hepa1-6 tumor mice control.

We employed non-targeted LC-MS/MS to identify significant alterations in metabolic processes in Hepa1-6 tumor-bearing mice tumor tissue treated with FZJDXJ therapy. Our results demonstrated marked differences in the overall metabolite profiles between the FZJDXJ and anti-PD-1 treatment groups compared to the model group (Figure 5A). As shown in Figures 5B, C, both interventions led to a growth in glucose metabolites within the tumors. Notably, glycolysis/gluconeogenesis of glucose metabolism emerged as pivotal metabolic pathways influenced by FZJDXJ in HCC treatment (Figure 5D). Finally, we revealed a notable increase in the expression of HK2, PFKPB3, PKM2, and GLUT1 by western blot in both the FZJDXJ and anti-PD-1 treatment compared to the model mice (Figure 5E). Furthermore, these protein expressions exhibited a positive correlation with remarkable CD8^+^T cell exhaustion phenotype and functionality (Figure 5F). These data suggest that FZJDXJ can potentially ameliorate CD8+T cell exhaustion by regulating the metabolic imbalance, particularly in the glycolytic pathway, within the HCC tumor microenvironment.

### 3.7 FZJDXJ activates the glycolytic metabolism of CD8+T cells *in vitro*

**Figure 6:**
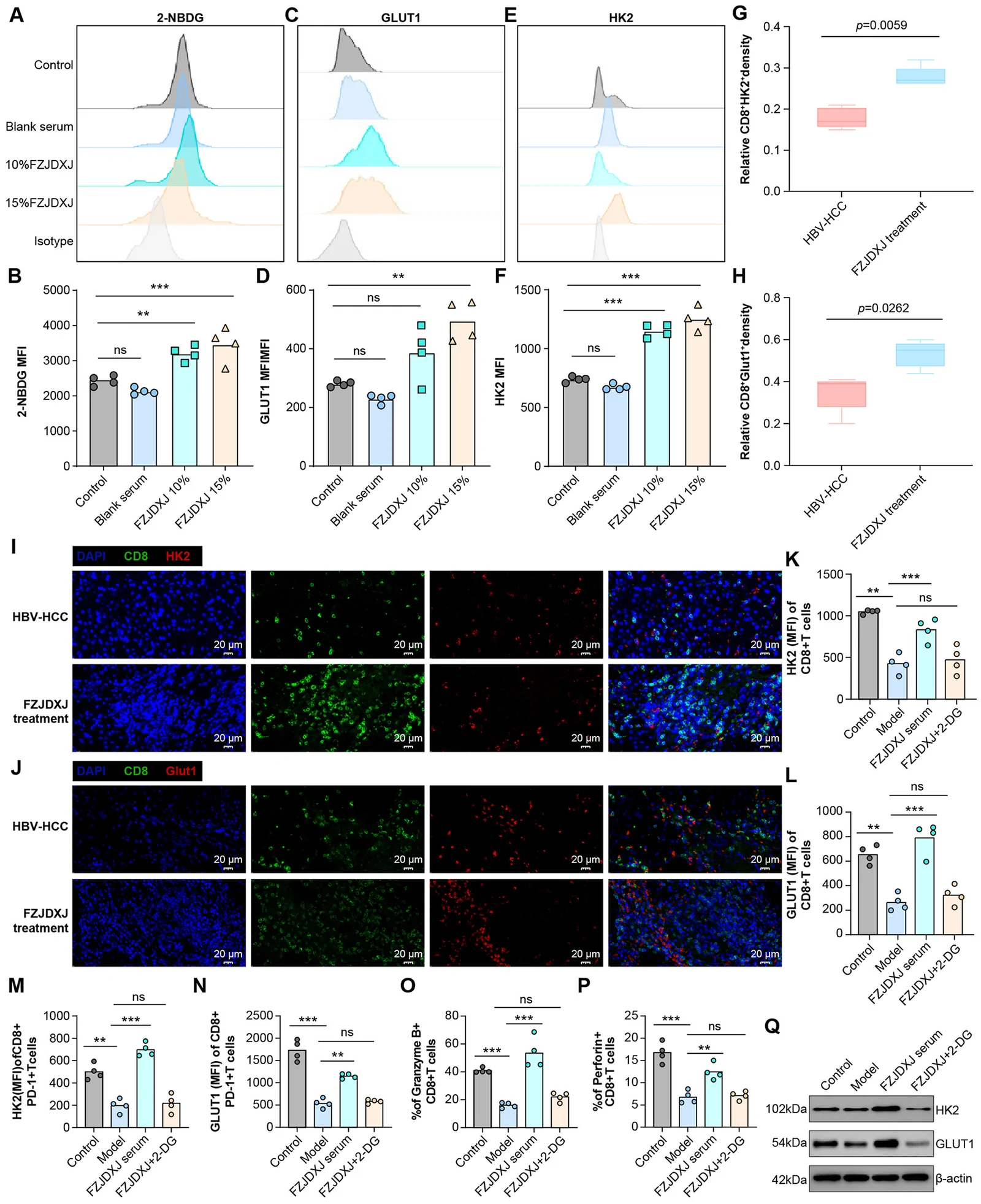
FZJDXJ activates the glycolytic metabolism of CD8^+^T cells. Flow cytometry analyzed the average fluorescence intensity (MFI) of glycolytic metabolic pathway key molecules on CD8+T cells isolated from mice tumor tissue with FZJDXJ containing serum stimulation *in vitro*. **(A–H)** CD8^+^ T cells were acquired by mouse auto-MACS Mitenyi CD8+T cell isolation kit from the tumor tissue of Hepa1-6 HCC mice. The cells were divided into control, blank serum, 10%, 15% FZJDXJ containing serum, and stimulated *in vitro* for 24 h. 2-NBDG **(A, B)**, GLUT1 **(C, D)**, HK2 **(E, F)** on CD8^+^T cell. GLUT1 and HK2 expression on CD8+ T cells in TME of HCC patients was measured by quantitative immunofluorescence, HK2, GLUT1 (red), and CD8 (green), and DAPI (blue) **(I–L)**. Flow cytometry and Western blot analysis of the glycolysis of CD8+T cells stimulated by 2-DG in FZJDXJ **(M, N, Q)** and the killing function of Granzyme B and Perforin **(O, P)**. All data are expressed as mean ± SD. *P < 0.05, **P < 0.01, ***P < 0.001.

Previous reports have shown that glucose competition between tumor cells and immune effector cells in the tumor microenvironment consumes necessities needed to maintain effector T cell function (18). It may be a driver of immunosuppression. Endowing CD8+T cells with glycolysis can improve the efficiency of immunotherapy and metabolic remodeling between tumor cells and T cells (19, 20). CD8+T cells were induced and activated IL-2, and anti-CD3/CD28 beads. After 3 days of induction, different proportion FZJDXJ and blank containing serum were added individually at the following concentrations, with phosphate-buffered saline (PBS) used as a control pyrimidine, we observed an increase in 2-NBDG, GLUT1, HK2 expression in the FZJDXJ group compared with that in the control group (*P* < 0.05; Figures 6A–F). Importantly, immunofluorescence detection of tumor tissue in HCC patients revealed that FZJDXJ can activate the expression of the first key rate limiting enzyme HK2 and glucose transporter GLUT1 in the microenvironment of CD8+T cell glycolysis metabolism pathway, fully verifying its ability to regulate the glycolysis metabolism of CD8+T cells and improve its immune response function (Figures 6G–J). We significantly blocked the upregulation of GLUT1 and HK2 by FZJDXJ in CD8+T cells *in vitro* with 2-DG (a glycolysis inhibitor), enhancing the cytotoxic function (secretion of Granzyme B and Perforin). This indicates that glycolysis is necessary for FZJDXJ to improve the immune response function of CD8+T cells. In summary, the comprehensive data obtained from clinical associations, mouse validation, and *in vitro* loss of function experiments now support a causal relationship: FZJDXJ enhances glycolysis, alleviates CD8+T cell exhaustion, and enhances anti-tumor immunity.

## 4 Discussion

Our comprehensive approach analyses unveiled the impact of FZJDXJ treatment on the anti-tumor immune phenotype and plasma metabolism in HCC patients. Additionally, we explored the causal relationship between significantly distinct metabolism profiles and anti-tumor immune responses. Furthermore, our multi-omics analysis encompassing clinical samples and HCC-bearing mouse models illuminated the effects of FZJDXJ on both the anti-tumor immune response and metabolism in HCC. This exploration enabled us to elucidate the underlying causal connections between different metabolic pathways and their influence on the anti-tumor immune response. The main findings are as follows: (1) Both FZJDXJ and ICI treatment alleviate the peripheral immune exhaustion phenotype in HBV-HCC patients to varying degrees, with CD8^+^ T cell regulation being the most notable effect. (2) FZJDXJ treatment exhibited specific characteristics in metabolomics and highlighted the intervention of metabolic pathways related to Glycolysis/gluconeogenesis, Central carbon metabolism in cancer, Fructose and mannose metabolism, Glycerophospholipid metabolis. (3) FZJDXJ exerts its anti-tumor effect by inhibiting tumor growth and alleviating CD8^+^ T cell exhaustion. Enhancing the glycolytic metabolic of CD8+T cells emerges as a potential therapeutic target.

Metabolic reprogramming plays a crucial role in T cell activation and differentiation, influencing T cell fate and immune response (21, 22). Prolonged exposure to hepatitis B virus (HBV) and tumor antigens progressively induces T cell exhaustion, characterized by distinct metabolic alterations in T cells and effector T cells. In normal immune effector cells, mitochondrial fatty acid oxidation (FAO) and oxidative phosphorylation (OXPHOS) are the primary mechanisms for energy acquisition (23, 24). In contrast, terminally exhausted T cells heavily rely on glycolytic metabolism, experiencing impairment in the glycolytic process, resulting in disruption of energy supply to T cells (6, 25). Therefore, it is imperative to explore novel mechanisms underlying T cell exhaustion, including factors related to metabolism or metabolic-associated epigenetic regulation, in order to identify potential therapeutic targets against T cell exhaustion (6). Our study has revealed distinct profiles of metabolites in HBV-HCC patients with FZJDXJ treatment. It clearly promoted robust activation of Glycolysis/gluconeogenesis. However, the specific impact of these physiological changes on the anti-tumor immune response in HCC remains less explored. Further investigation is warranted to elucidate the underlying mechanisms that underpin these roles. Our findings offer valuable insights into the potential mechanisms through which FZJDXJ modulates anti-tumor immune responses via metabolite pathways.

Currently, treatment options for patients with intermediate and advanced HBV-HCC primarily consist of systemic therapies. These systemic therapies predominantly involve anti-tumor strategies, including molecular targeted therapy, immunotherapy, chemotherapy, and TCM approaches (26). Furthermore, these treatment modalities also encompass the management of underlying HCC-associated conditions, such as antiviral therapy, hepatobiliary protection, and supportive symptomatic care. TCM is acknowledged for its multi-target and synergistic effects on HCC. Numerous clinical and preclinical studies have corroborated the effectiveness of TCM in HCC treatment (12, 27). Through various pathways and mechanisms, TCM can significantly impede the recurrence and metastasis of advanced HCC by targeting cancer stem cells, epithelial-mesenchymal transition, and effector T cells within the HCC tumor microenvironment (28, 29). FZJDXJ, an empirical formula employed in our hospital for many years, has demonstrated clear anti-HCC efficacy as evidenced by preliminary basic research (12). In this study, we initially observed that FZJDXJ treatment can ameliorate the immunosuppressive phenotype in patients with advanced HBV-HCC, as determined through analysis of a small clinical sample. Additionally, investigation into the impact of FZJDXJ on paired blood metabolites revealed heightened activity in Glycolysis/gluconeogenesis, Central carbon metabolism in cancer, Fructose and mannose metabolism pathways with advanced HBV-HCC, which was not observed with ICI treatment. Furthermore, a comparative analysis of metabolite differences demonstrated a positive correlation with the proportions of peripheral CD3^+^, CD4^+^ and CD8^+^ T cells while displaying a negative correlation with immunosuppressive CD8^+^ T and Treg cell phenotypes. These findings further indicate that FZJDXJ may alleviate the CD8+T cells immunosuppressive microenvironment in intermediate and advanced HBV-HCC patients by modulating the glycolysis metabolism pathways.

There are several limitations to this study. Firstly, it is important to note that the associations identified do not imply causation. We noted that this was a retrospective observational study with group assignment based on clinical treatment rather than randomization. The NLR imbalance did not alter the observed associations between FZJDXJ, glycolysis, and CD8+T cell exhaustion. Specifically, the metabolites that exhibited changes in mouse tumor tissue may affect different cell types. To further enhance our understanding of how glycolysis influences immune cells, we assessed the glycolytic activity of CD8^+^ T cells in HBV-HCC patients. Secondly, due to the COVID-19 pandemic, our access to sufficient clinical samples was restricted, thereby limiting the statistical power for analyzing the correlation between metabolites and the attenuation of anti-tumor immune suppression after FZJDXJ treatment. Future research should verify these findings in a large cohort, facilitating comprehensive measurements of metabolites. Lastly, our study cohort only examined the association between changes in glycolysis and CD8^+^ T cell exhaustion. The specific underlying mechanisms need to be further investigated *in vitro*.

In conclusion, FZJDXJ may activate the glycolytic metabolism rectify CD8+T cell immunosuppression to inhibit tumor progression. These insights indicate the potential of FZJDXJ as a targeted prescription to enhance anti-tumor immunity.

## Data Availability

The datasets presented in this study can be found in online repositories. The names of the repository/repositories and accession number(s) can be found in the article/Supplementary material.

## Supplementary material

Supplementary Figure S1 FZJDXJ impacts on CD4+ T cell exhaustion and differentiation of CD8+T cells. **(A)** Proportion of CD8+T cells. **(B)** CD8+T cell subtype estimation. **(C)** Proportion of CD4+T cells.

Supplementary Figure S2 Amino acid metabolites quantification. **(A–C)** Metabolic level estimation.

Supplementary Figure S3 Association of amino acids with immune cells. **(A–C)** Circle plot of the association between amino acids and immune cells.
