# Supplementary figures and images for: Natural product FZJDXJ enhances CD8+ T-cell glycolysis to relieve exhaustion and augment antitumor immunity in HBV+ hepatocellular carcinoma
Source: Front Nutr. 2026 Jun 12;13:1829200. doi: 10.3389/fnut.2026.1829200 (PMC13303808; doi:10.3389/fnut.2026.1829200)

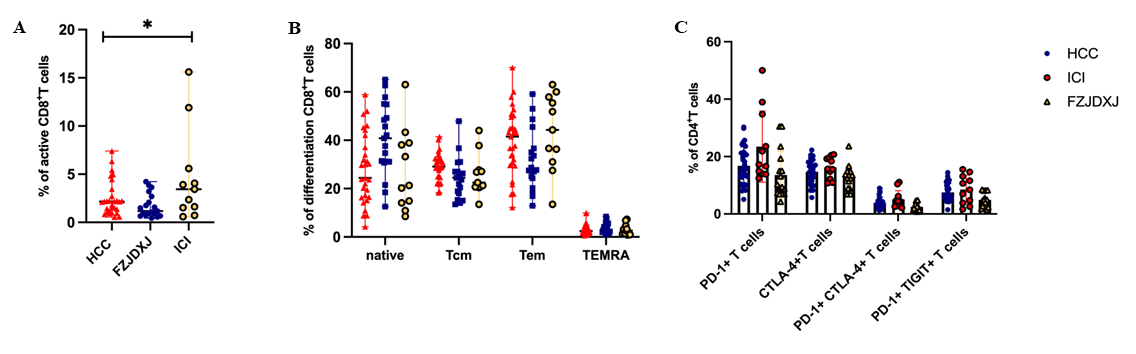

Supplement: Supplementary Figure S1 — FZJDXJ impacts on CD4+ T cell exhaustion and differentiation of CD8+T cells. (A) Proportion of CD8+T cells. (B) CD8+T cell subtype estimation. (C) Proportion of CD4+T cells. [file Image_1.tif]

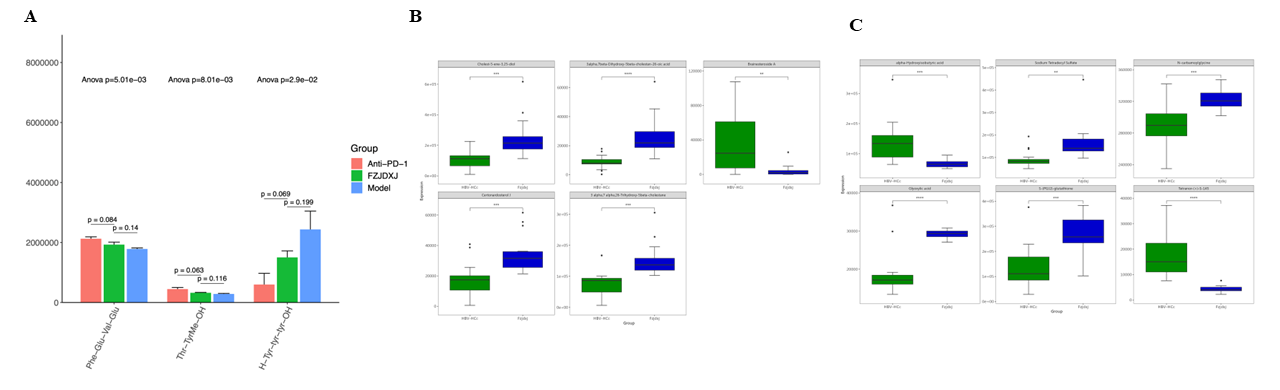

Supplement: Supplementary Figure S2 — Amino acid metabolites quantification. (A–C) Metabolic level estimation. [file Image_2.tif]

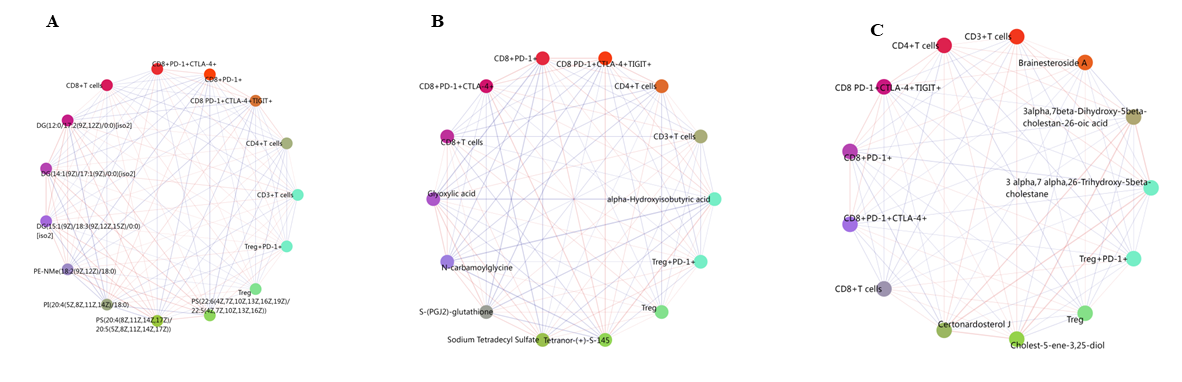

Supplement: Supplementary Figure S3 — Association of amino acids with immune cells. (A–C) Circle plot of the association between amino acids and immune cells. [file Image_3.tif]
